# Frozen emotions, frozen bodies: a systematic review of limbic system alterations in catatonia

**DOI:** 10.1007/s00406-026-02244-8

**Published:** 2026-04-24

**Authors:** Federica Marcolini, Valentina Beghelli, Damiano Tartarini, Valentina Di Giacomo, Silvia Tempia Valenta, Tomas Mastellari, Diana De Ronchi, Anna Rita Atti

**Affiliations:** 1 Department of Biomedical and Neuromotor Sciences, University of Bologna, Viale Pepoli 5, 40123 Bologna, Italy; 2 Department of Mental and Physical Health and Preventive Medicine, Università degli Studi della Campania “Luigi Vanvitelli”, Naples, Italy; 3 Doctoral Program of Global Health, Humanitarian Aid and Disaster Medicine, Vrije Universiteit Brussel, Brussels, Belgium; 4 Univ. Lille, Inserm, CHU Lille, U1172 - LilNCog - Lille Neuroscience & Cognition, F-59000 Lille, France

**Keywords:** Catatonia, Limbic system, Neuroimaging, Psychomotor disorders, Neural circuits

## Abstract

**Introduction:**

Catatonia is a complex neuropsychiatric syndrome characterized by psychomotor and affective symptoms. While current neurobiological models have focused on cortical and cerebellar dysfunctions, the limbic system—crucial for emotional regulation and motivation—remains comparatively underexplored. Emerging evidence suggests that limbic abnormalities may contribute to the pathophysiology of catatonia, offering a more integrated understanding of its clinical manifestations.

**Aims:**

This systematic review aims to synthesize current evidence on neurobiological abnormalities within the limbic system in individuals with catatonia and to identify which structures may be implicated in symptom development.

**Methods:**

Following PRISMA guidelines, systematic searches were conducted across MEDLINE, Web of Science, PsycINFO, and Scopus without date restrictions. Eligible studies included patients with a codified catatonia diagnosis undergoing instrumental or biological investigations involving limbic structures. Data extraction covered study design, diagnostic criteria, limbic regions assessed, and neuroanatomical or functional findings.

**Results:**

A total of 1792 studies were identified through database searching, of which 54 full-text articles were assessed; 14 studies met the inclusion criteria and were included. Six additional studies were identified through reference screening, resulting in 20 included articles: 13 observational studies and seven case reports. Most investigations highlighted alterations in the anterior cingulate cortex, including abnormal gyrification, grey matter reduction, and perfusion changes associated with catatonic symptoms. Orbitofrontal cortex abnormalities—though not classically part of the limbic system—were also frequently reported. Evidence regarding other regions was heterogeneous and included volumetric abnormalities of the amygdala and hypothalamus, as well as genetic or acquired factors affecting limbic biochemical regulation.

**Conclusions:**

This systematic review highlights the available neuroanatomical findings in patients with catatonia, supporting the involvement of limbic structures in the development and maintenance of this syndrome. However, variability in catatonia assessment, small sample sizes and study designs limit definitive conclusions. Future studies are needed to better understand the neural underpinnings of catatonia and inform treatment strategies.

## Introduction

Catatonia is a complex neuropsychiatric syndrome characterized by a set of motor, behavioral, and affective symptoms that combine in heterogeneous ways, producing diverse clinical pictures that might be difficult to recognize [1, 2].

The term “catatonia” was coined in 1874 by the German neuropsychiatrist Karl Ludwig Kahlbaum, who was the first to identify it as a clinical entity distinct from other psychoses known at the time. He described its core features, such as psychomotor agitation, fixed posturing, and affective disturbances, often accompanied by pathological withdrawal [3]. Throughout the twentieth century, catatonia was considered a subtype of schizophrenia; recently, catatonia has been identified as a syndrome associated with various psychiatric and medical conditions [4].

In the Diagnostic and Statistical Manual of Mental Disorders, Fifth Edition (DSM-5), catatonia is defined as a diagnostic specifier that may occur in three distinct contexts: in association with another mental disorder, due to a general medical condition, or in an unspecified form [5]. Catatonia is associated with psychiatric disorders in about 80% of cases, in particular mood disorders and schizophrenia. As for medical conditions, neurological, neurodegenerative, infectious, inflammatory or metabolic disorders are only some of the causes that can lead to catatonia [6, 7].

To diagnose catatonia, at least three of the following symptoms must be present simultaneously: stupor, catalepsy, waxy flexibility, mutism, negativism, posturing, mannerisms, stereotypies, agitation not influenced by external stimuli, grimacing, echolalia, and echopraxia [5]. The variability in the combination of these signs results in highly heterogeneous clinical presentations, often making diagnosis complex.

Catatonia’s clinical course is variable: it may present in acute forms, with a rapid onset (within days or weeks), or in a chronic form, with symptoms persisting for several weeks or even months, which is often associated with psychotic disorders and a less favorable prognosis [8, 9]. In rare cases, catatonia may progress to a malignant form, characterized by autonomic instability [10] and a mortality risk of up to 50% if not treated promptly [11, 12].

Treatment requires a dual approach: on the one hand, it is necessary to intervene on the catatonic state with specific treatments, such as benzodiazepines as first-line treatment, and electroconvulsive therapy (ECT) in refractory cases; on the other hand, it is essential to manage the underlying psychiatric disorder or medical condition [9, 13, 14]. Furthermore, it is essential to prevent complications, mainly related to immobility and withdrawal, through thromboprophylaxis, intravenous hydration, enteral nutrition and the use of specific aids to limit bedsores [14, 15].

In recent years, interest in catatonia has grown, particularly from a neurobiological research perspective [4, 16]. The first pathophysiological hypotheses involved dysfunction of motor cortical areas, the basal ganglia, and the cerebellum, providing an explanation for the motor symptoms typical of the syndrome [17–21]. However, these models fail to fully explain the affective and cognitive manifestations, which are frequently present. In this context, the limbic system has attracted increasing attention as a potential pathogenic hub, given its crucial role in emotion regulation, motivation, and higher-order cognitive processes [22, 23]. The limbic system can be broadly defined as a network of interconnected cortical and subcortical structures, including the hippocampus, amygdala, anterior and posterior cingulate cortex (ACC and PCC), parahippocampal gyrus, insula, orbitofrontal cortex (OFC), septal nuclei, mammillary bodies, and specific thalamic nuclei. These regions collectively support the processing of sensory information and the generation of emotional, autonomic, and behavioral responses [22, 24–26]. Their dysfunction may support the hypothesis of catatonia as a primitive fear syndrome [21], as well as explain some of the core features of catatonia, such as the Kraepelinian concept of 'paralysis of will' [27], now conceptualized as avolition and ambivalence.

Currently, the association between limbic system alterations and catatonia remains largely underexplored [18]. While recent systematic reviews have predominantly focused on cortical, cerebellar, and basal ganglia dysfunction in catatonia [18–20], a comprehensive synthesis specifically examining limbic system alterations across different investigative modalities has not yet been conducted. The limited evidence available can be attributed to the difficulty of investigating the deep and highly interconnected structures of the limbic system through imaging [28], as well as the scarcity of systematic studies in this area. A deeper examination of the limbic system’s role in the pathogenesis of catatonia could offer significant contribution, not only theoretically by expanding insight into the disorder’s underlying mechanisms, but also clinically by paving the way for more targeted treatment strategies and for alternative or integrated approaches [29].

In the clinical framework, clinicians may use different scales to evaluate catatonia and its related symptoms [30]; among the most commonly used are the Bush-Francis Catatonia Rating Scale [31], the Northoff Catatonia Rating Scale [32], and the DSM-5 checklist [33]. These instruments reflect a dual nature of the condition: on one hand, some items focus exclusively on motor symptoms of catatonia, assessing stupor, posturing, catalepsy, waxy flexibility, stereotypies, and akinesia; on the other hand, different items investigate the presence of affective symptoms such as anxiety, flat affect, affect incontinence, and impulsivity.

Based on these considerations, the present work aims to conduct a systematic review of the literature with the primary objective of identifying the main abnormalities of the limbic system in patients with catatonia, with particular attention to structural, functional, and molecular alterations identified through neuroimaging studies, post-mortem investigations and biochemical analyses. Secondly, this study will explore potential correlations between specific patterns of limbic dysfunction and the different clinical expressions of catatonia, in particular forms with a predominance of motor, affective or behavioral symptoms. Understanding the role of the limbic system in catatonia could help filling a significant knowledge gap and promote the development of more targeted therapeutic interventions.

## Methods

### Study design

This review was conducted in accordance with the methodological framework proposed by the Joanna Briggs Institute (JBI) for systematic reviews and was reported following the PRISMA (Preferred Reporting Items for Systematic Reviews and Meta-Analyses) guidelines [34]. The protocol for this study was registered in the International Prospective Register of Systematic Review (PROSPERO), with registration ID CRD42025646775.

### Eligibility criteria

We included studies investigating structural, functional, or molecular abnormalities using neuroimaging techniques (e.g., MRI, fMRI, PET), post-mortem analyses, or biochemical approaches.

We included all peer-reviewed articles published in English that reported on observational studies (including case–control, cohort, and cross-sectional designs), as well as experimental studies. Given the limited and heterogeneous evidence currently available on this emerging topic, case series and case reports were also considered to capture preliminary observations and underexplored aspects of the phenomenon. Studies were excluded if they were conducted on non-human subjects, or if they consisted of conference abstracts, editorials, or narrative reviews. We also excluded systematic reviews and meta-analyses, as well as any articles that did not specifically investigate or describe the limbic system in relation to catatonia.

### Search strategies

A literature search was conducted in the following electronic databases: PubMed/MEDLINE, Web of Science, PsycInfo and Scopus. The search strategy combined terms related to *catatonia* and the *limbic system*, including both MeSH terms and free-text keywords. In addition, the reference lists of included articles were manually screened to identify additional relevant studies. Two authors (FM and STV) independently executed the literature search, which was first launched on 15 February 2025 and subsequently updated on 15 October 2025 to capture any additional studies published after the initial search.

The search strategy gathered the results from the four databases and eliminated duplicates. Two researchers (DT and VDG) independently screened the articles by evaluating their titles and abstracts to assess the eligibility criteria, and disagreements were resolved through discussion with a third author (FM). Full-text articles were independently assessed by two reviewers (DT and VDG) in a double-blind manner. Any discrepancies in the evaluation were resolved through discussion or consultation with a third reviewer (FM). Only studies that met all predefined eligibility criteria were selected for inclusion.

### Data extraction

Data were extracted using a standardized charting form developed a priori. For each study, we collected information on the author(s), year of publication, and country of origin, as well as the study design and sample size. Details regarding the study population were also recorded, including age, diagnosis, and the diagnostic method used to identify catatonia. In addition, we documented the approaches employed to assess the limbic system—such as neuroimaging techniques or post-mortem analyses—and summarized the main findings related to structural, functional, or molecular alterations. Lastly, we extracted any reported associations between these neurobiological findings and the clinical manifestations of catatonia. Data extraction was performed independently by two reviewers (DT and VDG). The third author (FM) resolved any disagreements during the selection or extraction process.

### Synthesis of results

Given the heterogeneity of study designs and outcomes, a narrative approach was employed. Studies were grouped according to the specific limbic brain region investigated, regardless of whether the alteration was structural, functional, or molecular in nature.

### Risk of bias assessment

Two authors (VB and FM) independently evaluated the risk of bias for each included article. To reflect the profoundly different nature of these study types, we employed two separate assessment tools.

For the case reports, we used the JBI critical appraisal checklist for case reports [35]. For the cross-sectional studies, we used the Newcastle–Ottawa Scale (NOS) in its cohort-study version, applying the commonly used adaptation for cross-sectional designs [36]. In line with standard practice, items referring to follow-up (i.e., demonstration that the outcome was not present at baseline and adequacy of follow-up) were excluded, as they are not applicable to non-longitudinal studies [37–39]. Thus, we assessed three items in the Selection domain (max three stars), both items in the Comparability domain (max two stars), and only the item regarding the ascertainment of the outcome (max one star), resulting in a maximum achievable score of six stars.

## Results

### Study selection

**Fig. 1:**
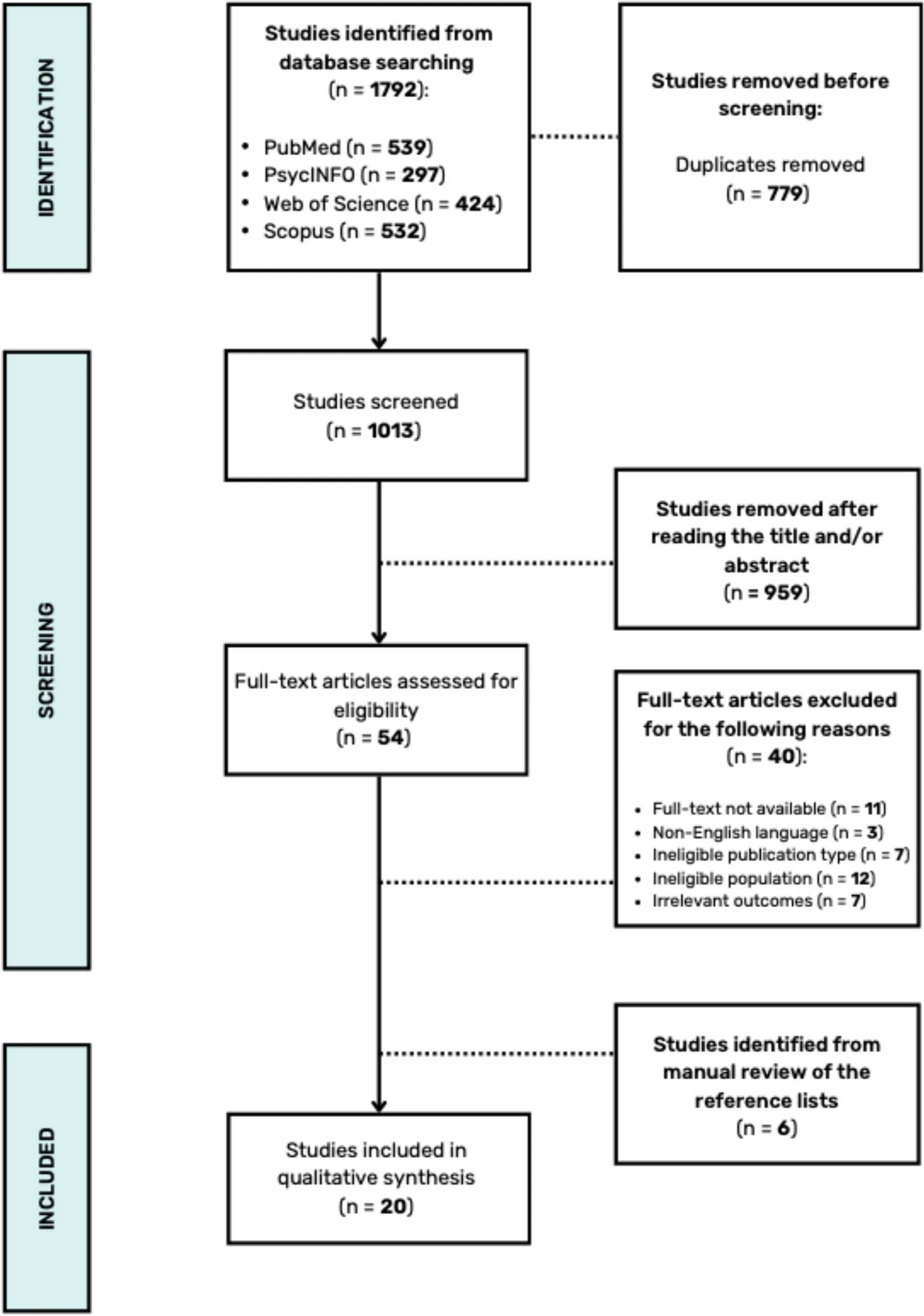
PRISMA flow diagram illustrating the study selection process. The diagram shows the number of records identified, screened, assessed for eligibility, and included in the final review, along with reasons for exclusions

Figure 1 shows the PRISMA flow diagram of the database search process. A total of 1,792 articles were retrieved from the four databases. After removing 779 duplicates, 1,013 records were screened based on title and abstract, resulting in the exclusion of 959 articles. The remaining 54 articles underwent full-text screening, after which 40 were excluded for not meeting the inclusion criteria. In addition to the database search, we performed a manual review of the reference lists of the included studies and identified six additional articles that met the eligibility criteria. In total, 20 articles were included in the final review.

### Description of the included studies

**Fig. 2:**
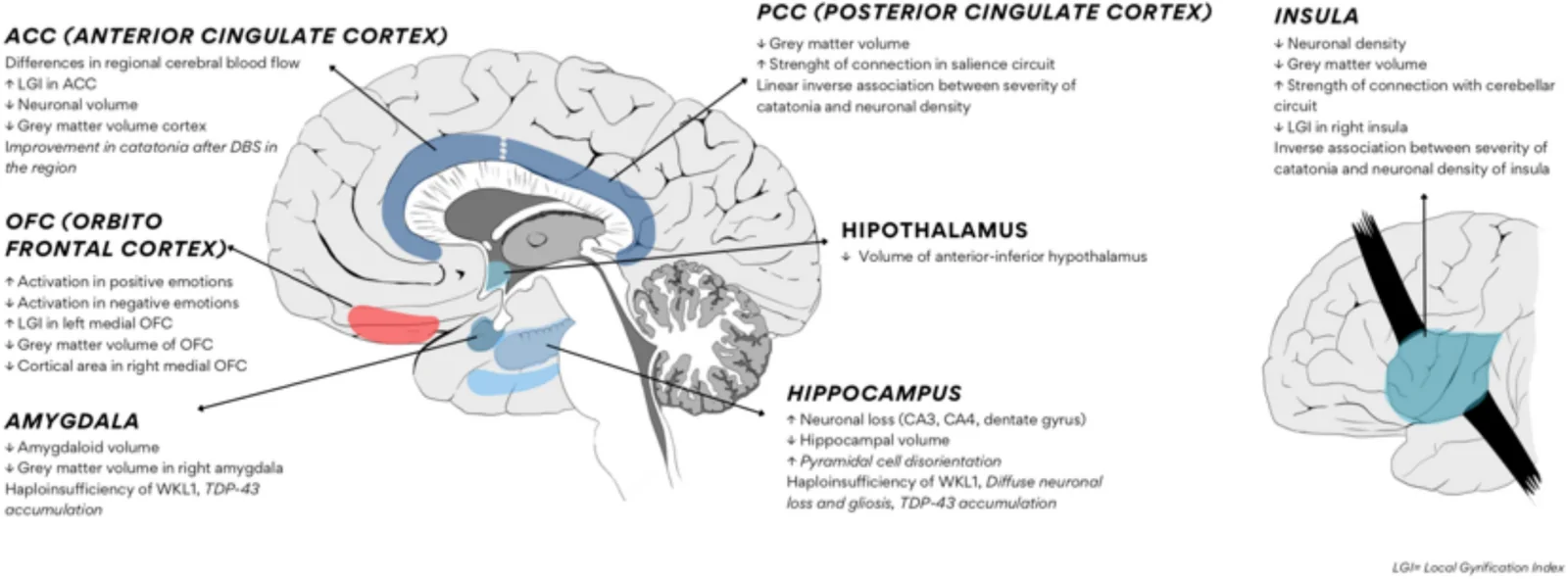
Visual summary of limbic system alterations in catatonia

20 articles were included (Table 1), all published between 1986 and 2024. The studies were conducted in Germany, Italy, France, Japan, India, U.S., U.K., Switzerland. Among the included studies, 12 were cross-sectional, one was a retrospective study, and the remaining seven were case reports, accounting respectively for 60%, 5%, and 35% of the total number of included publications. The number of enrolled patients ranged from one to 172, with an age span of the participants going from 18 to 65. All studies analyzed, either directly or indirectly, the anatomical, functional and structural relevance of certain brain regions in the illness of the patients, with a specific interest in catatonia. All findings regarding the limbic system were extrapolated from the included studies and subsequently organized into same-brain-region batches (Table 2). This clustering style was chosen in order to highlight the functional domains of the limbic system relating to catatonia. A visual summary of these regional associations is provided in Fig. 2.

**Table 1:** Summary of the 20 studies included in the review, divided in Observational Studies and Case Reports

Author(s), year	Country	Type of study	No; Age (y.o.); Sex (% M/F)	Diagnosis (criteria)	Scales used to evaluate Catatonia	Symptoms of catatonia considered	Part of Limbic system investigated	Assessment Method	Findings
Observational Studies									
Falkai and Bogerts, (1986)	Germany	Cross-sectional	SSD: 13; 43 y.o.; 15%/85% HC: 11; 43 y.o.; 64%/36%	SSD; Cat	ICD-9	–	Hippocampus	Histology	Neuronal loss in dentate gyrus: Cat > nonCat and Cat/Par Neuronal loss in CA3 e CA4: Cat/Par > Cat and nonCat
Gruzelier et al., (1988)	UK	Cross-sectional	SSD: 57; 33.7 y.o.; 53%/47% HC: 29; 33.7 y.o.; 55%/45%	SSD; Cat	DSM3	Cognitive	Hippocampus	Psychometric tests	Patients with catatonia show left temporal deficit but better frontal activity in psychometric tests performance
Kuroki N, (1998)	Japan	Cross-sectional	SSD: 28; 53.5 y.o.; 71%/29% HC: 20; 53.5 y.o.; 80%/20%	SSD (DSM4-TR)	DSM4-TR	–	Hippocampus	Histology	Pyramidal cell disorientation Cat > nonCat, though not statistically significant
Meyer et al., (2001)	Germany	Cross-sectional	SSD-Cat: 57; -; - HC: 80; -; -	SSD; Cat	–	–	Amygdala Hippocampus	Genetics	WKL1 haploinsufficiency is correlated to SSD-Cat in this sample WKL-1 is markedly expressed in the amygdala, caudate nucleus, hippocampus and thalamus
Devaney JM, (2002)	U.S	Cross-sectional	SSD: 33; -; 70%/30% HC: 1; -; -	SSD; Cat	–	–	Amygdala Hippocampus	Genetics	WLK1 cannot be said to have a causal role in the generation of catatonia in this sample
Northoff G, (2004)	Germany	Cross-sectional	SSD: 10; 41.6 y.o.; 50%/50% HC: 10; 41.6 y.o.; 50%/50%	SSD; Cat Bipolar disorder; Cat	BFCRS NCRS	Motor and affective	ACC PCC OFC	Functional MR	OFC activation in negative and positive emotions is different between SSD-Cat vs SSD-nonCat (< activation in negative emotions, > activation in positive emotions) OFC as a potentially catatonia-related zone
Walther et al., (2017)	Switzerland	Cross-sectional	SSD-Cat: 15; 37.6 y.o.; 73%/27% SSD-nonCat: 27; 37.6 y.o.; 63%/37% HC: 41; 37.6 y.o.; 61%/39%	SSD (DSM5)	BFCRS	Motor and affective	Insula ACC	MR	rCBF in ACG Cat ≠ nonCat Neuronal density in the insula and PFC Cat < nonCat Inverse linear correlation between severity of catatonia and neuronal density in the insula and PFC
Hirjak et al., (2019)	Germany	Cross-sectional	SSD-Cat: 59; > 18 and < 65 y.o.; 53%/47% SSD-nonCat: 22; > 18 and < 65 y.o.; 50%/50%	SSD (DSM4-TR)	DSM4-TR NCRS	Motor and behavioral	ACC OFC Insula	MR	Cortical surface (mm^2^): SSD-Cat < SSD-nonCat in SPG, right OFC, LOG LGI in ACC and medial OFC: SSD-Cat > SSD-nonCat LGI in left STG, right ITG and insula: SSD-Cat < SSD-nonCat
Dean et al., (2020)	U.S	Cross-sectional	SSD-nonCat: 43; > 16 and < 65 y.o.; 81%/19% SSD-Cat: 43; > 16 and < 65 y.o.; 74%/26% HC: 86; > 16 and < 65 y.o.; 74%/26%	SSD (DSM4-TR); Cat and nonCat	BFCRS	–	ACC Insula	Psychometric tests	Verbal fluency and processing speed: SSD-Cat < SSD-nonCat Motor symptoms circuit and whole-brain analysis: SSD-Cat = SSD-nonCat. A difference is found in the ACG and Insula when comparing HC to the entire SSD group (Cat and nonCat)
Parekh et al., (2021)	India	Cross-sectional	Cat: 15; 26.3 y.o.; 47%/53% HC: 15; 26.3 y.o.; 47%/53%	Cat	BFCRS DSM5	Motor	Insula PCC	MR	Strength of cerebellar circuit connection to the insula: Cat > HC Strength of PCC connection in the salience network: Cat < HC
Moyal et al., (2023)	France	Retrospective	SSD-Cat: 58; 36 ± 17 y.o.; 69%/31% SSD-nonCat: 65; 37 ± 13 y.o.; 68%/32% HC: 82; 29 ± 8 y.o.; 48%/52%	SSD (DSM5); Cat and nonCat	BFCRS DSM5	Motor and affective	OFC	MR	Type III OFC sulcal pattern: SSD-Cat > HC Type III OFC sulcal pattern: SSD-cat > SSD-nonCat Type I and II OFC sulcal pattern: SSD-Cat < HC and SSD-nonCat Type I OFC sulcal pattern: SSD-nonCat < HC
Fritze et al., (2024)	Germany	Cross-sectional	SSD-Cat: 30; > 18 and < 65 y.o.; 53%/47% SSD-nonCat: 28; > 18 and < 65 y.o.; 46%/54% HC: 20; > 18 and < 65 y.o.; 50%/50%	SSD (DSM4-TR); Cat and nonCat	NCRS	Motor and affective	Amygdala Hippocampus Hypothalamus	MR	Volume of anterior-inferior hypothalamus: SSD-Cat < SSD-nonCat Dimensions of amygdala, hippocampus ad hypothalamus: SSD Cat < HC and SSD-nonCat Volume of amygdala’s nuclei associated to a severity of affective symptoms on NCRS and anxiety item in BRPS, but fails to resist multiple-comparison correction First study that mentions anterior-inferior hypothalamus role
Walther S, (2024)	Switzerland	Cross-sectional	98 SSD and 42 HC; 36.3 y.o.; 50%/50%	SSD (DSM5)	BFCRS DSM5 NCRS	Motor and affective	Amygdala ACC PCC OFC	MR	Neuronal density in the insula, PFC, OFC, right amygdala and ACC: Cat < HC

Author(s), year	Country	No; Age (y.o.); Sex (% M/F)	Diagnosis (criteria)	Scales used to evaluate Catatonia	Symptoms of catatonia considered	Part of Limbic system investigated	Imaging if present	Findings
Case Reports								
Lim et al., (1986)	U.S	3; 55–67 y.o.; 100% M	Cat during prolonged seizure	–	Motor	–	EEG	Reported cases of epilepsy with a catatonic presentation
Narayanaswamy et al., (2012)	India	1; 40 y.o.; 100% F	SSD; Cat	BFCRS	Motor	CSP	CT	First reported case of CSP in SDD-Cat
Yasaki et al., (2013)	Japan	1; 66 y.o.; 100% F	Unspecified Somatic Symptom and Related Disorder (DSM5)	DSM5	Motor	CSP Cavum Vergae	MR	First reported case of CSP with CV in SDD-Cat
Watanabe et al., (2017)	Japan	1; 58 y.o.; 100% M	Cat and FTLD-TDP type C	DSM5	Motor and behavioral	Hippocampus Parahippocampal gyrus Amygdala Cingulate gyrus	MR Histology	First reported case with histological evidence of neuronal involvement of FTLD in Cat Diffused cerebral atrophy, with TDP-43 deposits in limbic areas Notified cases of catatonia in FTD with C9ORF72 amplification
Endres et al., (2020)	Germany	1; 40 y.o.; 100% M	Probable Encephalitis; Cat	DSM5	Motor	Hippocampus	FDG-PET	Reported case of catatonia with autoantibody against cilia of granular cells without encephalitis criteria on PET exam
Minghetti et al., (2021)	Italy	1; 18 y.o.; 100% F	Functional Neurological Symptom Disorder	–	Motor	ACC	MR PET	Prevalently motor symptom catatonia successfully treated with DBS in ACC
Zuckemberg et al., (2024)	U.S	1; 29 y.o.; 100% M	SSD; Cat	DSM5	Motor	CSP	CT	Reported case of CSP with Schizophrenia with catatonia

Note. *ACC* Anterior Cingulate Cortex; *BFCRS* Bush-Francis Catatonia Rating Scale; Cat, patient with Catatonia; *CSP* Cavum Septum Pellucidum; *CT* Computerized Tomography; *DSM5* The Diagnostic and Statistical Manual of Mental Disorders, 3th Edition; *DSM4-TR* The Diagnostic and Statistical Manual of Mental Disorders, 4th Edition—Text Revision; *DSM5* The Diagnostic and Statistical Manual of Mental Disorders, 5th Edition; *EEG* electroencephalogram; *FTLD-TDP* Frontotemporal Lobar Degeneration with TDP-43 pathology; *HC* Healthy Control; *ICD9* International Classification of Disease, 9th edition; ITG, Inferior Temporal Gyrus; LGI, Local Gyrification Index; *LOG* Lateral Occipital Gyrus; *M* male; MR, Magnetic Resonance; No, Number of patients; *NCRS* Northoff Catatonia Rating Scale; nonCat, patient without Catatonia; *OFC* Orbitofrontal Cortex; *FDG-PET* fluorodeoxyglucose-positron emission tomography; *PCC* Posterior Cingulate Cortex; PFC, Prefrontal Cortex; *SPG* Superior Parietal Gyrus; *SSD* Schizophrenia Spectrum Disorder; STG, Superior Temporal Gyrus; *TDP-43* TAR DNA-binding Protein 43 kDa; y.o., years old

**Table 2:** Overview of the limbic regions identified across the included studies

Brain region	Type of study	Author and year	Findings
Hippocampus	CS	Falkai and Bogerts, (1986)	Neuronal loss in CA4 e dentate gyrus: Cat > nonCat and Cat/Par
			Neuronal loss in CA3 e CA4: Cat/Par > Cat and nonCat
	CS	Gruzelier et al., (1988)	In psychometric tests, catatonic patients have left temporal deficits and better frontal performance; the hippocampus is assessed indirectly through tests that are assumed to reflect its functionality
	CS	Kuroki N, (1998)	Pyramidal cell disorientation Cat > nonCat, though not statistically robust
	CS	Meyer et al., (2001)	Haploinsufficiency of WKL1 is associated to SSD-Cat in this sample
			WKL1 is markedly expressed in limbic areas, including the hippocampus
	CS	Devaney et al., (2002)	WKL1 cannot be said to have a causal role in catatonia in the sample analysed in this study
	CS	Fritze et al., (2024)	Hippocampal volume (mm^3^) SSD-Cat < SSD-nonCat and HC
	CR	Watanabe et al., (2017)	Diffuse neuronal loss and gliosis is found in hippocampus cortex of a Cat
			Moderate amount of TDP-43 accumulation in hippocampus
	CR	Endres et al., (2020)	Reported case of Cat with autoantibody against cilia of granular cells without encephalitis criteria on PET exam
ACC	CS	Walther et al., (2017)	rCBF in ACC of Cat ≠ nonCat
	CS	Hirjak et al., (2019)	LGI in SSD-Cat > SSD-nonCat in ACC
	CS	Dean et al., (2020)	Neuronal volume in ACC of SSD-Cat and SSD-nonCat < HC
	CS	Walther S, (2024)	Grey matter volume cortex of ACC in Cat < HC
	CR	Minghetti et al., (2021)	Cat with motor symptom in remission after the patient received DBS in ACC
Amygdala	CS	Meyer et al., (2001)	Haploinsufficiency of WKL1 is associated to SSD-Cat in this sample
			WKL1 is markedly expressed in some limbic areas, including the amygdala
	CS	Devaney et al., (2002)	WKL1 cannot be said to have a causal role in catatonia in the sample analysed in this study
	CS	Fritze et al., (2024)	Amygdala’s volume (mm^3^) in SSD-Cat < SSD-nonCat and HC
			Associations between amygdala nuclei volumes and NCRS affective scores and BPRS item #2 (reflecting anxiety). However, these correlations did not survive correction for multiple comparisons and showed only trend level associations
	CS	Walther S, (2024)	Grey matter volume in right amygdala: Cat < HC
	CR	Watanabe et al., (2017)	Moderate amount of TDP-43 accumulation in amygdala
Insula	CS	Walther et al., (2017)	Neuronal density of insula in Cat < nonCat
			Linear inverse association between severity of catatonia and neuronal density of insula
	CS	Hirjak et al., (2019)	LGI SSD-Cat < SSD-nonCat in right insula
	CS	Dean et al., (2020)	Grey matter volume of the insula in SSD-Cat and SSD-nonCat < HC
	CS	Parekh et al., (2021)	Strength of connection in cerebellar circuit with the insula in Cat > HC
PCC	CS	Walther S, (2024)	Grey matter volume of PCC in Cat < HC
	CS	Parekh et al., (2021)	Strength of connection in salience circuit with PCC in Cat > HC
			Linear inverse association between severity of catatonia and neuronal density of PCC
OFC	CS	Northoff G, (2004)	OFC activation in negative and positive emotions SSD-Cat vs SSD-nonCat (< activation in negative emotions, > activation in positive emotions)
			OFC as potentially catatonia-related zone
	CS	Hirjak et al., (2019)	LGI SSD-Cat > SSD-nonCat in left medial OFC
			Cortical area in right medial OFS in SSD-Cat < SSD-nonCat
	CS	Walther S, (2024)	Grey matter volume of OFC in Cat < HC
CSP	CR	Narayanaswamy et al., (2012)	Case of CSP and SSD-Cat; no measures are given
	CR	Yasaki et al., (2013)	Case of CV variant of CSP in SSD-Cat
	CR	Zuckemberg et al., (2024)	Case of CSP in SSD-Cat
Cingulate gyrus	CR	Watanabe et al., (2017)	Moderate amount of TDP-43 accumulation in cingulate gyrus
Parahippocampal gyrus	CR	Watanabe et al., (2017)	Moderate amount of TDP-43 accumulation in parahippocampal gyrus
Hypothalamus	CS	Fritze et al., (2024)	Volume (mm^3^) of anterior-inferior hypothalamus of SSD-Cat < SSD-nonCat and HC

Note: *ACC* Anterior Cingulate Cortex; *BFCRS* Bush-Francis Catatonia Rating Scale; Cat, patient with Catatonia; Cat/Par, patient with either Catatonic Schizophrenia or Paranoid Schizophrenia; *CR* Case-Report; *CS* Cross-Sectional; *CSP* Cavum Septum Pellucidum; *CV* Cavum Vergae; *DBS* Deep Brain Stimulation; *HC* Healthy Control; *LGI* Local Gyrification Index; *NCRS* Northoff Catatonia Rating Scale; nonCat, patient without catatonia; *OFC* Orbitofrontal Cortex; *PET* positron emission tomography; *PCC* posterior cingulate cortex; PFC, Prefrontal Cortex; rCBF, regional Cerebral Blood Flow; *SSD* schizophrenia spectrum disorder; *TDP-43* TAR DNA-binding Protein 43 kDa

*Hippocampus*: The hippocampus is one of the central structures of the limbic system and it is responsible for memory processing, learning, spatial navigation, and emotions [40]. Considering its role in catatonia, several studies offer insights, although sometimes indirectly. Early post-mortem investigations by Falkai and Bogerts highlighted neuronal loss in specific hippocampal subregions (CA3, CA4, and dentate gyrus) in schizophrenia patients with catatonic features, particularly those with a paranoid component [41]. Kuroki’s group also observed disorganization of pyramidal cells in catatonia, but their findings lacked robust statistical confidence [42]. Indirect evidence comes from Gruzelier et al., who noted left temporal lobe deficits in psychometric tests in catatonic individuals, suggesting potential hippocampal involvement [43]. A more recent study, conducted by Fritze and colleagues, reported reduced hippocampal volume in patients with a diagnosis of Schizophrenia Spectrum Disorder (SSD) with catatonia compared to the same type of patients without catatonia and healthy controls [44]. Genetic links have also emerged, with Meyer and colleagues identifying WKL1 haploinsufficiency, a gene expressed in limbic areas including the hippocampus, in a sample of catatonic SSD patients [45]. Furthermore, case reports have contributed to the understanding: Watanabe and his research group described diffuse neuronal loss, gliosis, and TDP-43 accumulation in the hippocampus of a catatonic patient, while Endres and colleagues presented a case of autoantibodies against granular cell cilia without typical encephalitis criteria, raising questions about purely psychiatric forms of encephalitis and potential hippocampal involvement [46, 47].

*Amygdala*: Located in the anterior medial temporal lobe, the amygdala acts as pivotal player in saliency detection, modulating activity across widespread brain networks, shaping adaptive behavior [48]. This structure has also been implicated in the presentation of catatonia through both genetic and structural investigations. Meyer and colleagues identified a connection between WKL1 haploinsufficiency and SSD with Catatonia, noting that WKL1 is markedly expressed in various limbic regions, including the amygdala [45]. Subsequent volumetric studies have reinforced this link: Fritze and colleagues reported a reduction in amygdala volume in SSD patients with catatonia compared to both SSD individuals without catatonia and healthy controls [44]. While Fritze also explored potential associations between amygdala nuclei volumes and affective scores or anxiety, these correlations, though trending, did not withstand correction for multiple comparisons. This observation aligns with a more recent finding by Walther and colleagues, who independently reported decreased right amygdala gray matter volume in catatonia relative to healthy controls [49]. Furthermore, a case report by Watanabe and colleagues revealed the presence of moderate TDP-43 accumulation within the amygdala of a catatonic patient, suggesting potential neuropathological underpinnings in this region [47].

*Anterior cingulate cortex*: The ACC shares connections with other limbic areas, including the amygdala and OFC, involved in emotion and reward-related processing [22]. The ACC has garnered considerable attention in the investigation of catatonia, with several studies pointing to its potential involvement. Alterations in regional cerebral blood flow within the ACC distinguish catatonic patients from their non-catatonic counterparts, as noted by Walther et al. [50]. Further structural differences have been observed, with Hirjak and colleagues reporting increased local gyrification index in the rostral ACC of individuals with SSD and catatonia compared to those with SSD but no catatonia [51]. Moreover, a consistent finding across different studies indicates a reduction in ACC gray matter volume in catatonic patients when compared to healthy controls [49, 52]. Beyond these cross-sectional observations, a compelling case report by Minghetti and colleagues documented the successful resolution of catatonic motor symptoms following deep brain stimulation targeting the ACC, suggesting a potential modulatory role of this region in the pathophysiology of catatonia [53].

*Posterior cingulate cortex*: The PCC, while supporting functions such as spatial processing and memory, is directly involved in the limbic network through back projections from the hippocampal system and inputs from the orbitofrontal cortex [22]. The PCC has also been identified as a region of interest in catatonia, with research highlighting both structural alterations and changes in functional connectivity. A recent study by Walther and colleagues reported a reduction in gray matter volume within the PCC of catatonic patients when compared to healthy controls, suggesting a structural deficit in this region [49]. Complementing this, Parekh and colleagues investigated functional connectivity and found increased strength of connection within the salience network involving the PCC in catatonic individuals relative to healthy controls [54]. Intriguingly, their work also identified an inverse linear association between catatonia severity and neuronal density in the PCC, hinting at a potential direct link between microstructural integrity in this area and the clinical expression of catatonic symptoms.

*Insula*: The insular cortex belongs to an elaborate neural circuitry connecting the higher cortex, the limbic structures, basal ganglia, and autonomic system [55]. Its specific functions are still poorly understood, but there is strong evidence pointing to a role in the broader limbic network with emerging data describing also a contribution in high-level cognitive functions [56]. It has emerged as a significant area of interest in understanding catatonia. Investigations into its role have revealed both structural and functional alterations. Walther and colleagues observed reduced neuronal density in the insula of catatonic patients compared to non-catatonic individuals, and notably, this reduction showed an inverse linear association with catatonia severity [50]. Further structural distinctions were highlighted by Hirjak and colleagues, who reported a decreased local gyrification index in the right insula of patients with SSD and catatonia compared to those with SSD but no catatonia [51]. Broadening this finding, Dean and colleagues demonstrated that grey matter volume in the insula was generally reduced in all SSD patients, regardless of the presence of catatonia, when compared with healthy controls, suggesting a broader involvement in SSD [52]. Beyond structural changes, functional connectivity studies, such as that by Parekh and colleagues, have identified increased strength of connection within the cerebellar-insular circuit in catatonic patients relative to healthy controls, pointing to altered network dynamics involving this crucial region [54].

*Orbitofrontal Cortex*: In the last decade, the OFC has gathered special attention by the neuroscientific literature as a critical region for emotion regulation and decision-making. It has also been implicated in the neurobiology of catatonia through a combination of functional and structural studies. Northoff and colleagues observed distinct patterns of OFC activation in individuals with SSD and catatonia compared to those without catatonia during emotional processing when patients were exposed to specific stimuli [57]. Specifically, catatonic patients with SSD showed reduced activation and increased deactivation for negative emotions, alongside increased activation and reduced deactivation for positive emotions, suggesting the OFC as a potentially affectivity-related area in catatonia. Further investigations into structural differences by Hirjak and colleagues revealed increased local gyrification index in the left medial OFC of SSD patients with catatonia, while the cortical surface area in the right medial OFC was smaller in this group compared to SSD without catatonia [51]. More recently, Walther and colleagues reported a general reduction in OFC grey matter volume in catatonic patients compared to healthy controls, reinforcing the notion of structural abnormalities in this region in catatonia [49]. Of note is the work done by Moyal and colleagues, focusing on the sulcal patterns of the OFC, in which the authors report a higher prevalence of type III pattern on the left hemisphere amongst schizophrenic patients with catatonia compared to those without catatonia [58].

*Other regions*: Beyond the more extensively studied limbic structures, nascent evidence points to the involvement of several other encephalic regions in catatonia, warranting further investigation. The cingulate and parahippocampal gyri, both integral to limbic circuitry, have shown signs of neuropathological changes, as evidenced by moderate TDP-43 accumulation in these regions in a case of catatonia [47]. Similarly, structural differences in the hypothalamus, a critical regulator of numerous physiological functions, have been identified; Fritze and colleagues reported reduced volume of the antero-inferior hypothalamus in SSD catatonic patients when compared to both non catatonic SSD patients and healthy controls [44]. Furthermore, the presence of a Cavum Septum Pellucidum (CSP) has been repeatedly noted in case reports involving schizophrenia with catatonia, though these observations primarily highlight co-occurrence without providing specific metrics of association with catatonia specifically [59–61]. While these findings from diverse methodologies are preliminary, they collectively suggest a broader involvement in catatonia of other brain regions, inviting more systematic and comprehensive research into their specific contributions to the syndrome's pathophysiology.

### Clinical correlation with limbic structures alterations

Our secondary objective was to explore potential associations between specific limbic abnormalities and equally specific clinical facets of catatonia. Amongst all the studies included in our work, none of them had a degree of detail high enough to warrant a clear correlation between certain kinds of symptoms and brain structures. It is therefore imperative that, aside from disclosing the lack of results achieved regarding our secondary objective, this absence be considered as a lead for future investigations.

### Risk of bias

**Fig. 3:**
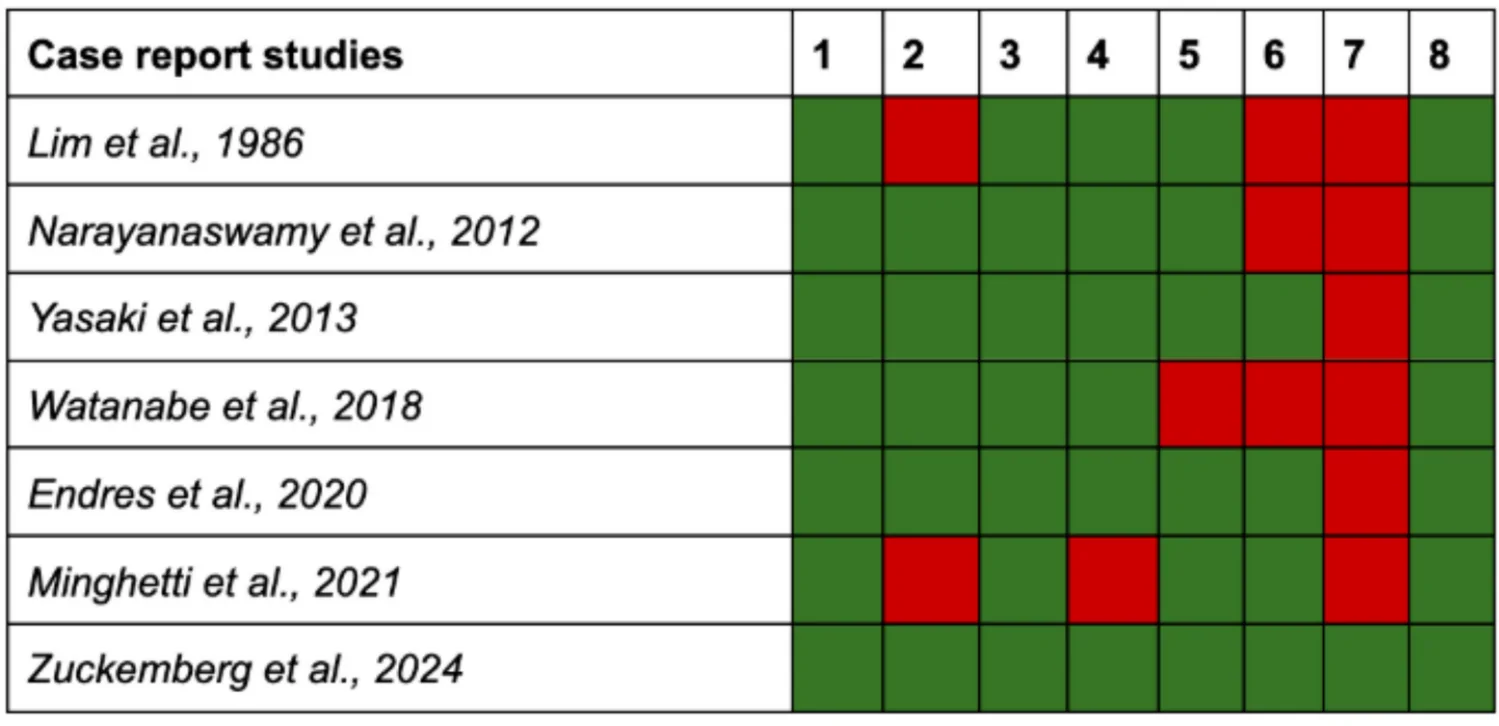
Quality assessment of the case report studies according to the JBI Critical Appraisal tools for Systematic reviews, Checklist for Case Reports. Note. Green, criterion adequately met; Red, criterion not met

**Fig. 4:**
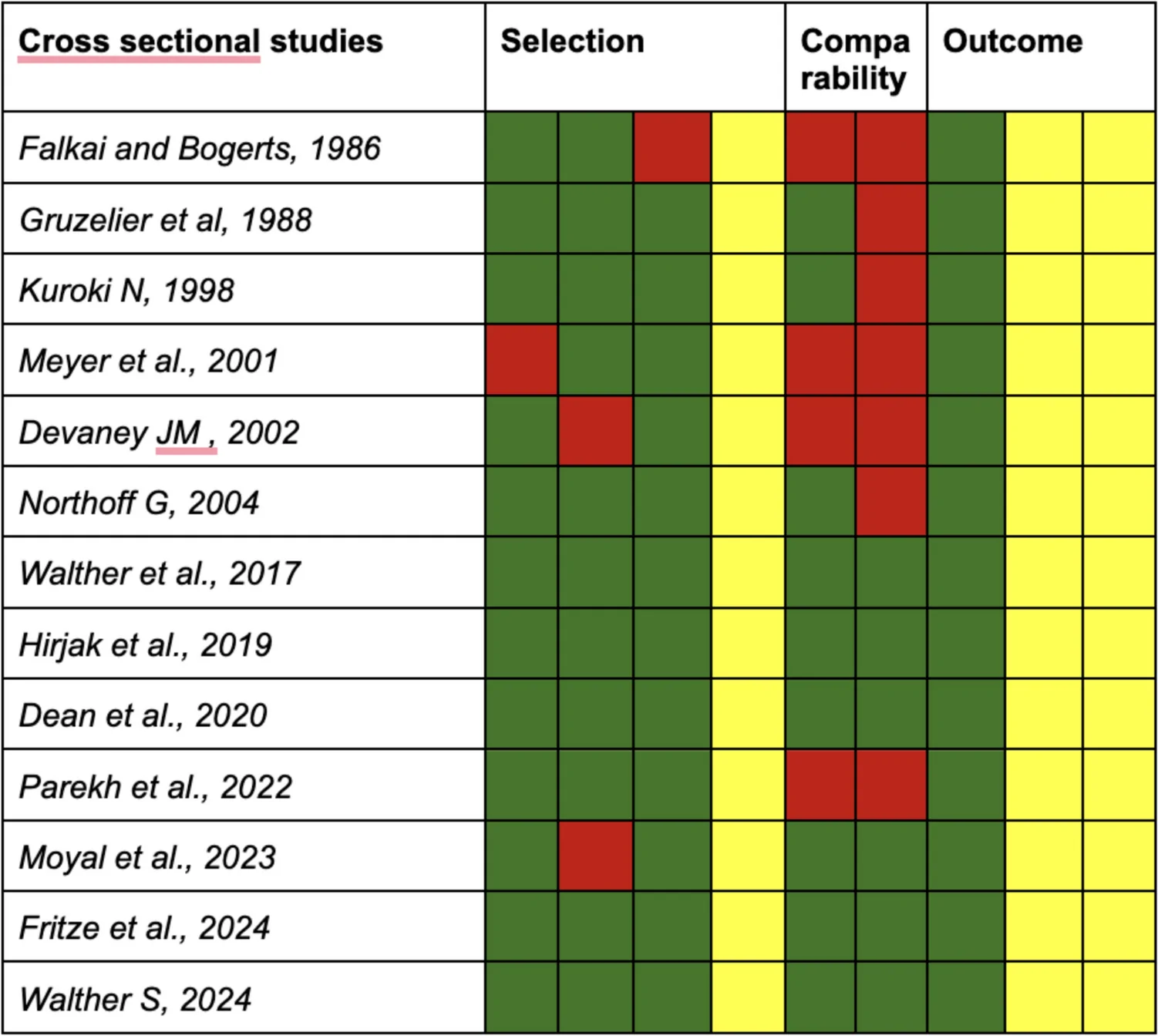
Quality evaluation of the cross-sectional studies included in the review according to the NOS for Cohort Studies. Note. Green, criterion adequately met; Red, criterion not met; Yellow, criterion not applicable due to study design

Figures 3 and 4 show the risk of bias across included studies. Among the case reports, 14.3% (n = 1) achieved a full score (8/8 items), 28.6% (n = 2) scored 7/8 (87.5%), 14.3% (n = 1) scored 6/8 (75%), and 42.8% (n = 3) scored 5/8 (62.5%) according to the JBI Critical Appraisal Checklist for Case Reports. Overall, most case reports provided clear clinical descriptions and diagnostic reasoning, although incomplete reporting of patient history and differential diagnoses was observed in several cases. The cross-sectional studies were evaluated using an adapted version of the NOS for Cohort Studies, as detailed in the Methods. Most studies demonstrated adequate participant selection procedures and clearly defined comparison groups. However, variability was noted in the control of confounding, with several studies lacking statistical adjustment or matching procedures. Outcome ascertainment was also inconsistently standardized, with some studies not employing validated assessment tools.

## Discussion

The present review provides converging evidence that catatonia is not adequately explained as a primarily motor syndrome. Rather, the findings point to a multidimensional disorder rooted in altered limbic network dynamics, in which circuits responsible for affective modulation, salience attribution, and interoceptive integration fail to interact effectively with cortical systems governing behavioral control. This interpretation aligns with contemporary neuropsychiatric frameworks conceptualizing catatonia as a state of *network dysregulation* emerging from disrupted coordination between subcortical emotional systems and higher-order regulatory processes [13, 62]. Recent work has similarly emphasized the role of affective dysfunction and fronto-limbic imbalance in the pathophysiology of catatonia, despite the relative scarcity of direct investigations of limbic structures [18, 21, 63, 64].

Across the included studies, abnormalities within several limbic structures were consistently observed.

The hippocampus and amygdala, key structures in emotional memory, motivational drive, and threat response, displayed structural reductions (neuronal loss in early post-mortem investigations and reduced volume in more recent neuroimaging studies) and neuropathological markers (WKL1 haploinsufficiency, pathological protein accumulation) in individuals with catatonia. These findings suggest a vulnerability within circuits governing emotional salience and contextual appraisal. This aligns with clinical phenomenology: catatonia frequently arises in states of overwhelming affect, anxiety, or psychosis, where emotional processing exceeds regulatory capacity [65, 66]. From this perspective, catatonia may represent a maladaptive shutdown response, a “freeze state” emerging when affective load surpasses integrative tolerance thresholds [67].

Alterations in the ACC and PCC further support this interpretation. The ACC, involved in conflict monitoring and motivational regulation, displayed gray matter reductions and morphological abnormalities specifically associated with catatonic presentations, and the resolution of catatonic motor symptoms following deep brain stimulation targeting this region offers a compelling, though preliminary, suggestion of a modulatory role. The PCC, a key hub of the default mode network, demonstrated reduced neural integrity and altered functional connectivity, with severity-dependent associations. Together, these findings suggest disrupted integration between internal emotional states and external behavioral adaptation, consistent with theories of catatonia as a disorder of internal–external contextual mapping [10, 68].

The insula, central to interoceptive awareness and emotional embodiment, showed both structural and connectivity abnormalities (reductions in neuronal density, alterations in gyrification, and increased cerebellar-insular connectivity) in catatonic patients. Given that catatonia often involves diminished bodily responsiveness and altered awareness of one’s own motor state, insular disruptions may underlie the characteristic detachment or “disconnection” frequently described clinically [69–71]. These findings align with models positing impaired integration of interoceptive states and emotional salience in catatonia [66].

Finally, OFC—central to emotional valuation and decision-making—showed differences in gyrification, cortical surface area, gray matter volume, and activation patterns during emotional processing tasks. Abnormalities in this area reinforce the hypothesis of dysregulated affective valuation and impaired cognitive control over emotional processes: if affective signals cannot be prioritized, inhibited, or contextualized appropriately, behavioral output may collapse into rigidity, withdrawal, or psychomotor shutdown [72, 73]. Right medial OFC volume has also revealed to be a predictor of benzodiazepine response in catatonic patients, with larger volumes associated with response [74].

Additional evidence points toward alterations in the hypothalamus, parahippocampal regions, and structural anomalies such as the cavum septum pellucidum, though these findings are more preliminary and require systematic validation.

Rather than representing isolated regional abnormalities, these findings can be integrated within a circuit-based model of limbic–cortical interactions, in which specific nodes function as dynamic “switches” regulating behavioral output [75].Within this framework, alterations in systems responsible for affective salience, interoceptive processing, and higher-order regulatory control converge to impair the translation of internal states into adaptive motor behavior. Dysfunction within this network may result in a failure of integration between emotional processing and action, ultimately leading to behavioral withdrawal and motor inhibition characteristic of catatonia. This circuit-based interpretation aligns with psychomotor models of catatonia, which conceptualize the syndrome as arising from dysfunction in distributed networks linking affective, cognitive, and motor domains [62, 76]. In this perspective, limbic dysfunction represents a core component of a broader system in which emotional and interoceptive signals influence motor output. Taken together, these findings support a model in which catatonia reflects a disturbance in the balance between limbic affective drive and cortical regulatory control: when emotional or interoceptive signals exceed integrative capacity, the system may default to behavioral withdrawal and motor inhibition, resembling a high-intensity freeze response [75]. This framework provides a coherent neurobiological explanation for the occurrence of catatonia across diverse psychiatric and medical conditions and is consistent with its evolutionary interpretation as a defensive response to extreme internal or external threat [72, 73, 77]. This provides a unifying framework linking limbic dysfunction to the psychomotor expression of catatonia.

The abnormalities identified across limbic regions in this review could help clarify specific components of the brain network involved in catatonia. As for OFC dysfunction, it could be hypothesized that it disrupts top-down GABAergic modulation of the basal ganglia and shifts activity toward the ACC, suppressing its motor subdivision and contributing to akinesia and mutism [76, 78]. Upstream, amygdala hyperactivation – which could be sustained by the volumetric reductions and their association with affective symptom severity – may prompt overwhelming emotional arousal that overrides prefrontal regulatory circuits, producing the tonic immobility characteristic of akinetic catatonia [79]. Reduced grey matter volume does not necessarily imply reduced functional activity. In fact, smaller volume may reflect reduced inhibitory interneuron density, which would paradoxically result in disinhibited output from the amygdala. Furthermore, hippocampal pathology may further dysregulate default mode network modulation of the sensorimotor system [80], while hypothalamic involvement plausibly underlies autonomic instability in malignant forms. The resting-state data reported by Parekh et al. [54] confirm that alterations in the ACC and the insula might be functionally responsible for the uncoupling of these two areas from the salience network, while aberrantly causing them to short-circuit with the motor network in acute catatonia.

The secondary aim of this review, assessing associations between specific limbic abnormalities and distinct clinical phenotypes, could not be addressed due to insufficient granularity in the available data. Rather, it highlights a critical methodological gap: the field lacks systematic studies that integrate neurobiological measures with clearly defined catatonic subtypes (motor-predominant, affective-predominant, stuporous vs. excited states). The absence of such correlations therefore reflects a methodological limitation rather than a negative finding and underscores the need for studies combining multimodal neuroimaging with systematic clinical subtyping. Addressing this gap is likely to transform our mechanistic understanding of the syndrome.

### Strengths and limitations

This review presents several methodological and conceptual strengths. It integrates evidence derived from diverse investigative domains, including neuroimaging, post-mortem neuropathology, structural morphometry, connectivity analyses, and genetic findings, thereby minimizing the risk that the recurrent implication of limbic regions results from a modality-specific bias. The focus on a neurobiological dimension that has historically received limited attention in catatonia research provides conceptual advancement, offering a coherent framework that bridges clinical phenomenology with neural circuit dysfunction. The convergence of findings across heterogeneous methods, despite small sample sizes, supports the emerging view of catatonia as a disorder of limbic-cortical network regulation rather than a purely motor phenomenon.

Nevertheless, the conclusions of this review must be interpreted with caution due to several important limitations. First, the available evidence base is quantitatively and qualitatively constrained: most studies involved small samples, lacked replication, and were often limited to single-center designs or case reports, which markedly restricts generalizability. Second, substantial heterogeneity exists in diagnostic criteria, inclusion thresholds, and measurement tools. Third, methodological discrepancies across imaging protocols further limit comparability and may obscure subtle convergent effects. Equally critical is the paucity of detailed symptom characterization. Few studies stratified patients according to catatonic subtype or dominant symptom dimension (motor, affective, or behavioral), preventing any meaningful exploration of structure-phenotype relationships. This gap impedes the formulation of mechanistic hypotheses linking neural alterations to clinical expression. The included studies are also characterized by marked variability in the representation of acute, chronic, and malignant forms of catatonia, which may differentially influence neurobiological findings. In particular, chronic presentations may allow more stable neuroimaging assessment compared to acute states, introducing potential bias in the observed patterns. Moreover, the cross-sectional nature of nearly all studies precludes temporal inference regarding causality or state dependence, leaving open the question of whether observed limbic abnormalities represent trait vulnerabilities, epiphenomena of illness severity, or effects of pharmacological exposure.

## Conclusion

The evidence synthesized in this review supports a reconceptualization of catatonia as a disorder arising from dysfunction within limbic–cortical networks, rather than as a syndrome defined solely by motor abnormalities. This perspective may also help explain the transdiagnostic nature of catatonia across psychiatric and medical conditions. Structural, functional, and neuropathological alterations across the hippocampus, amygdala, cingulate cortex, insula, and orbitofrontal regions indicate that disruptions in affective regulation, salience processing, and interoceptive integration play a central role in the expression of catatonic states. Interpreted within a circuit-based framework, these alterations suggest that catatonia may emerge from impaired coordination between emotion-generating systems and higher-order regulatory regions, leading to maladaptive behavioral output, including motor inhibition. This perspective, consistent with contemporary psychomotor models, bridges clinical phenomenology with underlying circuit dysfunction and establishes a foundation for mechanistically informed diagnostic and therapeutic strategies. Future large-scale, multimodal, and longitudinal studies integrating standardized diagnostic procedures, comprehensive symptom profiling, systematic characterization of treatment response, and harmonized imaging protocols will be crucial to elucidate the causal and functional significance of limbic abnormalities in catatonia, as well as to establish reliable outcome measures, thereby advancing neurobiological understanding and improving clinical care. Methodologically, future studies would benefit from advanced neuroimaging approaches, including ultra-high field MRI for improved anatomical precision, magnetic resonance spectroscopy to characterize neurochemical alterations, and underutilized techniques such as diffusion tensor imaging and susceptibility-weighted imaging [81–83], which may further clarify the role of limbic circuitry in catatonia.

## Data Availability

No datasets were generated or analysed during the current study.
